# Do robot-related complications influence 1 year reoperations and other clinical outcomes after robot-assisted lumbar arthrodesis? A multicenter assessment of 320 patients

**DOI:** 10.1186/s13018-021-02452-z

**Published:** 2021-05-12

**Authors:** Nathan J. Lee, Ian A. Buchanan, Venkat Boddapati, Justin Mathew, Gerard Marciano, Paul J. Park, Eric Leung, Avery L. Buchholz, John Pollina, Ehsan Jazini, Colin Haines, Thomas C. Schuler, Christopher R. Good, Joseph M. Lombardi, Ronald A. Lehman

**Affiliations:** 1 Department of Orthopaedics, Columbia University Medical Center, The Och Spine Hospital at New York-Presbyterian, 161 Fort Washington Avenue, New York, NY 10032 USA; 2 Department of Neurosurgery, University of Virginia Health System, Charlottesville, VA USA; 3 Department of Neurosurgery, State University of New York, Buffalo, NY USA; 4 Department of Orthopaedics, Virginia Spine Institute, Reston, VA USA

**Keywords:** Robot-assisted spine surgery, Lumbar fusion, Mazor X, Complications, Reoperations

## Abstract

**Background:**

Robot-assisted platforms in spine surgery have rapidly developed into an attractive technology for both the surgeon and patient. Although current literature is promising, more clinical data is needed. The purpose of this paper is to determine the effect of robot-related complications on clinical outcomes

**Methods:**

This multicenter study included adult (≥18 years old) patients who underwent robot-assisted lumbar fusion surgery from 2012-2019. The minimum follow-up was 1 year after surgery. Both bivariate and multivariate analyses were performed to determine if robot-related factors were associated with reoperation within 1 year after primary surgery.

**Results:**

A total of 320 patients were included in this study. The mean (standard deviation) Charlson Comorbidity Index was 1.2 (1.2) and 52.5% of patients were female. Intraoperative robot complications occurred in 3.4% of patients and included intraoperative exchange of screw (0.9%), robot abandonment (2.5%), and return to the operating room for screw exchange (1.3%). The 1-year reoperation rate was 4.4%. Robot factors, including robot time per screw, open vs. percutaneous, and robot system, were not statistically different between those who required revision surgery and those who did not (*P*>0.05). Patients with robot complications were more likely to have prolonged length of hospital stay and blood transfusion, but were not at higher risk for 1-year reoperations. The most common reasons for reoperation were wound complications (2.2%) and persistent symptoms due to inadequate decompression (1.5%). In the multivariate analysis, robot related factors and complications were not independent risk factors for 1-year reoperations.

**Conclusion:**

This is the largest multicenter study to focus on robot-assisted lumbar fusion outcomes. Our findings demonstrate that 1-year reoperation rates are low and do not appear to be influenced by robot-related factors and complications; however, robot-related complications may increase the risk for greater blood loss requiring a blood transfusion and longer length of stay.

## Introduction

Robotic surgery has rapidly developed into an attractive option for spine surgeons as evidenced by the growing literature in the last few years [1–4]. Numerous reports demonstrate excellent pedicle screw accuracy and early studies have explored the impact of robot-assisted spine surgery on reducing radiation exposure, length of hospital stay, operative time, and perioperative complications in comparison to conventional freehand technique [5–10]. However, current literature has been derived from mostly single-center or single-surgeon studies [11–15]. Furthermore, the impact of robot-related complications on clinical outcomes remains unclear.

Reoperation rates are particularly important in spine surgery because they imply disease progression or surgical complications. Complete reoperation data in robotic spine surgery remains to be elucidated as most studies only report intraoperative revisions due to screw misplacement and rates are exceedingly low. In a large, retrospective study of 359 patients, Keric et al. reported a reoperation rate of 1.7% [8]. Other authors in smaller, prospective randomized control trials have reported reoperation rates of 0.0% at up to 2 years [14, 16]. Additional reported post-operative complications in robotic surgery that have led to revision surgery include wound healing issues, new neurological deficits, and infections [7, 8].

The purpose of this study is to examine the influence of robot-related complications on clinical outcomes, including 1-year reoperations, after robot-assisted lumbar fusion. To the authors’ knowledge, this is the first multicenter assessment of complications related to robot-assisted lumbar fusion with a minimum 1-year follow-up. We hypothesize that robot-related factors do not influence reoperation risk, but may prolong operative time. Our study’s findings may further contribute to the acceptance of robotic-assisted spine surgery as an excellent surgical option for surgeons and patients alike.

## Materials and Methods

### Patient selection

We reviewed a multicenter database of adult patients (≥18 years old) who underwent robot-assisted lumbar arthrodesis between 2012 and 2019. All surgeries were performed by either the Renaissance (Renaissance, Mazor Robotics Ltd., Israel) or Mazor X system (Mazor Robotics Ltd.). Patients with missing data or who underwent spinal fusion at unrelated levels (cervical, thoracic) were excluded from our study. The minimum follow-up was 1 year after the date of surgery. This study was approved by the institutional review board.

### Data collection

Several perioperative factors were collected and included patient demographics, comorbidity profile, smoking status, body mass index, and primary preoperative diagnosis. Robot factors included open vs. percutaneous surgery, robot time spent per screw, robot screws placed per patient, and robot system (Renaissance or Mazor X). Other operative factors included primary vs. revision surgery, number of instrumented levels per patient, pelvic fixation, and total operative time.

Robot-related complications included intraoperative exchange of robot screw due to breach, robot abandonment due to registration issues or unreachable anatomy, intraoperative loss of motor/sensory function, and return to the operating room during the same inpatient stay for screw removal and/or exchange. Even though surgeons generally would not characterize an intraoperative redirection of a screw using freehand, flouro-assist or navigation as a “complication,” we wanted to have strict criteria when assessing any variance with the planned surgery. We evaluated the impact of each robot-related factor on reoperation risk as well as the composite of all robot-related complications, which was defined as the variable, “any robot-related complication.” A reoperation was defined as any return to the operating room during a subsequent hospital encounter. In other words, a return to the operating room during the same index hospital stay was not considered a reoperation.

### Data analysis

The primary outcome of our study was reoperation within 1 year after the index surgery. Secondary outcomes of interest included estimated blood loss, perioperative blood transfusion, total operative time, and length of hospital stay. The chi-square test and *t* test were used to compare categorical and continuous variables, respectively. Both bivariate and multivariate logistic regression analyses were performed to determine if robot-related complications were associated with reoperation within 1 year after primary surgery. Statistical significance was defined as a *P* value <0.05. SAS Studio Version 3.4 (SAS Institute Inc., Cary, NC) was used for all statistical analyses.

## Results

A total of 320 patients were included in this study. The mean (standard deviation) Charlson Comorbidity Index (CCI) was 1.2 (1.2), 44.7% (*N*=143) of patients were obese, 10% (*N*=32) were prior/current smokers, and 52.5% (*N*=168) of patients were female. The most common preoperative primary diagnoses included high-grade spondylolisthesis (60.3%, *N*=193), degenerative disk disease (18.1%, *N*=58), and spinal stenosis (9.7%, *N*=31). The mean number of instrumented levels was 2.8 (0.9), 17.5% (*N*=56) of surgeries were open (vs. percutaneous), 48.8% (*N*=156) were performed with the X robot (vs. Renaissance), 6.6% (*N*=21) of patients had pelvic fixation, the mean robot time was 28.7 (21.3) minutes, and the mean total operative time was 126 (92) minutes (Table 1).

**Table 1:** Bivariate analysis of post-discharge 1 year reoperations for patient, operative, and robot-related factors

	All		No reoperation		Reoperation		
	N	%	N	%	N	%	***P*** value
**Total # of patients**	320		306	95.6%	14	4.4%	
**Female**	168	52.5%	159	52.0%	9	64.3%	0.367
**Obese (BMI>30 kg/m**^**2**^**)**	143	44.7%	134	43.8%	9	64.3%	0.132
**CCI, mean (standard deviation, SD)**	1.2 (1.2)		1.2 (1.2)		1.4 (1.2)		0.398
**Prior/current smoker**	32	10.0%	31	10.1%	1	7.1%	0.716
**Preoperative diagnosis**							
High-grade spondylolisthesis	193	60.3%	184	60.1%	9	64.3%	0.877
Degenerative disk disease	58	18.1%	56	18.3%	2	14.3%	
Spinal stenosis	31	9.7%	30	9.8%	1	7.1%	
Degenerative scoliosis	24	7.5%	22	7.2%	2	14.3%	
Pseudarthrosis	10	3.1%	10	3.3%	0	0.0%	
Other	4	1.3%	4	1.3%	0	0.0%	
**Robot factors**							
Open (vs. percutaneous)	56	17.5%	54	17.6%	2	14.3%	0.746
Robot time per patient (minutes), mean (SD)	28.7 (21.3)		28.5 (21.5)		32.2 (20.3)		0.599
Robot screws per patient, mean (SD)	5.1 (3.4)		5.0 (3.4)		5.7 (3.4)		0.462
Robot time per screw (minutes/screw), mean (SD)	6.6 (3.8)		6.6 (3.9)		6.1 (1.9)		0.668
Robot system							
Renaissance	164	51.3%	160	52.3%	4	28.6%	0.103
X	156	48.8%	146	47.7%	10	71.4%	
**Other operative factors**							
Prior spine surgery	27	8.4%	26	8.5%	1	7.1%	0.856
Instrumented levels per patient, mean (SD)	2.8 (0.9)		2.8 (0.9)		3.2 (1.4)		0.272
Pelvic fixation	21	6.6%	19	6.2%	2	14.3%	0.233
Operative time (minutes), mean (SD)	126 (92)		126 (94)		129 (58)		0.924
**Any intraoperative robot-related complication (patients have ≥1 complication)**	11	3.4%	10	3.3%	1	7.1%	0.437
Exchange of malpositioned robot screw	3	0.9%	3	1.0%	0	0.0%	0.710
Robot abandonment	8	2.5%	8	2.6%	0	0.0%	0.540
Due to registration error	3	0.9%	3	1.0%	0	0.0%	0.710
Due to unreachable anatomy	1	0.3%	1	0.3%	0	0.0%	0.830
Other	4	1.3%	4	1.3%	0	0.0%	0.667
Intraoperative dural tear	0	0.0%	0	0.0%	0	0.0%	
Intraoperative loss of motor/sensory function	0	0.0%	0	0.0%	0	0.0%	
Return to operating room during same inpatient stay for screw removal and/or exchange	4	1.3%	3	1.0%	1	7.1%	**0.042**
**Other non-robot-related complications**							
Intraoperative dural tear	3	0.9%	3	1.0%	0	0.0%	0.710

Intraoperative robot complications occurred in 3.4% (*N*=11) of patients. These included robot abandonment (2.5%, *N*=8), return to the operating room during the same inpatient stay for screw removal and/or exchange (1.3%, *N*=4), and intraoperative exchange of screw for breach (0.9%, *N*=3). No intraoperative loss of motor/sensory function was observed in this study’s cohort. Intraoperative dural tear occurred in 3 patients (0.9%), but these occurred during decompression during revision laminectomy and were not directly related to robot factors (Table 1).

The 1-year reoperation rate was 4.4% (*N*=14) [1 to 90 days = 2.5%, *N*=8; 91 days to 1 year = 1.9%, *N*=6]. Robot characteristics were not statistically different between those who required revision surgery and those who did not (*P*>0.05). These factors included open surgery [vs. percutaneous] (no reoperation = 17.6%, *N*=54 vs. reoperation = 14.3%, *N*=2), robot time (seconds) per screw (no reoperation = 6.6 vs. reoperation = 6.1), and robot system (Renaissance: no reoperation = 52.3%, *N*=160 vs. reoperation = 28.6%, *N*=4; Mazor X: no reoperation = 47.7%, *N*=146 vs. reoperation = 71.4%, *N*=10). Intraoperative robot complications such as exchange of a breached screw and robot abandonment for either registration errors or unreachable anatomy did not appear to influence reoperation risk. Interestingly, return to the operating room during the same inpatient stay for screw removal and/or exchange was a significant risk factor for reoperation (no reoperation 1.0% (*N*=3) vs. reoperation 7.1% (*N*=1)) (*P*=0.042) in the bivariate analysis (Table 1).

In the secondary outcome analysis, patients with robot complications were more likely to have prolonged length of hospital stay (any robot complication: 5.9 days vs. no robot complication: 4.8 days, *P*=0.019), a higher estimated blood loss (any robot complication: 324 mL vs. no robot complication: 78 mL, *P*<0.001), and an increased blood transfusion rate (any robot complication: 21.4% vs. no robot complication: 1%, *P*<0.001). Robot complications did not appear to be associated with longer operative time or robot time (Table 2).

**Table 2:** Bivariate analysis of clinical outcomes and any robot-related complication during the index surgery

	All	No robot complication	Any robot complication	***P*** value
**Perioperative blood transfusion, # of patients (%)**	6 (1.9%)	3 (1.0%)	3 (21.4%)	**<0.001**
**Estimated blood loss (mL), mean (SD)**	87 (146)	78 (122)	324 (392)	**<0.001**
**Robot time (minutes), mean (SD)**	28.7 (21.3)	28 (21)	45 (40)	0.357
**Operative time (minutes), mean (SD)**	126 (92)	124 (90)	171 (146)	0.189
**Length of hospital stay (days), mean (SD)**	4.8 (1.5)	4.8 (1.5)	5.9 (1.9)	**0.019**
**Any reoperation within 1 year after surgery**	11 (3.4%)	10 (3.3%)	1 (7.1%)	0.437

The most common reasons for reoperation were wound complications (2.2%, *N*=7) and persistent symptoms due to inadequate decompression (1.5%, *N*=5). In this study cohort, no reoperations were due to screw malposition or implant failure (Table 3). In the multivariate analysis for 1-year reoperations, robot-related factors and any robot-related complications were not independent risk factors (Table 4).

**Table 3:** Reasons for reoperation within 1 year after robot-assisted lumbar fusion

	N	%
**1 to 90 days after index surgery**		
Any	8	2.5%
Wound complication	6	1.9%
Persistent symptoms due to inadequate decompression	2	0.6%
Implant failure	0	0.0%
Dura fistula	0	0.0%
Screw malposition	0	0.0%
**91 days to 1 year after index surgery**		
Any	6	1.9%
Persistent symptoms due to inadequate decompression	3	0.9%
Wound complication	1	0.3%
Proximal junctional kyphosis	1	0.3%
Adjacent segment disease	1	0.3%
Implant failure	0	0.0%
Dura fistula	0	0.0%
Screw malposition	0	0.0%

**Table 4:** The risk factors for any reoperation within 1 year after robot-assisted lumbar fusion based on multivariate logistic regression

Risk factors	Adjusted odds ratio	95% Confidence interval		***P*** value
**Patient**				
Female	1.9	0.4	8.6	0.424
Obese	12.2	1.3	116	**0.029**
CCI	1.2	0.6	2.3	0.578
Smoker	<0.01	<0.01	>999	
**Preoperative diagnosis** (reference = degenerative disk disease)				
Degenerative scoliosis	6.8	0.4	124	0.916
High-grade spondylolisthesis	1.4	0.14	13.3	0.949
Pseudarthrosis	<0.01	<0.01	>999	
Spinal stenosis	0.2	0	26.2	0.991
Other	<0.01	<0.01	>999	
**Robot operative factors**				
Open vs. percutaneous	0.2	0.01	6.1	0.347
Total number of robot screws	1.2	0.7	2.2	0.556
Robot time/screw (minutes/screw)	0.9	0.7	1.2	0.393
**Other operative factors**				
Prior spine surgery	1.3	0.1	14.4	0.856
Total instrumented levels	0.5	0.1	2.7	0.419
Pelvic fixation	153	1.1	>999	**0.045**
Total operative time	1	1	1	0.903
Estimated blood loss	1	1	1	0.908
**LOS**	1.2	0.8	1.8	0.477
**Any robot-related complication**	<0.01	<0.01	>999	

## Discussion

Over the last two decades, there has been an increasing amount of literature in support for robot-assisted spine surgery. Most of this literature has focused on pedicle screw accuracy, and recent meta-analyses on randomized controlled trials have found robot-guided spine cases to be more accurate in pedicle screw placement with fewer proximal facet violations than conventional freehand techniques [17, 18]. However, other intraoperative robot complications, such as robot abandonment due to registration issues, are important to identify since they may have a negative impact on operative time and complications; however, their true incidence is not known since they are rarely mentioned in current literature. Furthermore, the consequence of robot-related complications on clinical outcomes, including post-discharge reoperations, has not been examined.

Using the Renaissance robot from 2011 to 2016, Keric et al. examined the outcomes for 406 patients who underwent thoracolumbar robot-assisted spine surgery [8]. Intraoperative robot complications included conversion to freehand due to registration failure (1.7%, *N*=7), dural tears (6.4%, *N*=26), and screw misplacement requiring revision surgery (0.48%, *N*=2 screws). The registration issues were seen in patients with severe osteopenia and obese patients whose significant soft tissue resulted in poor radiographic quality. All dural tears occurred during decompression or cage implantation. Screw breach most often occurred due to skiving or platform displacements. The overall revision rate was 9.4%, and included wound complications (4.9%, *N*=20), durotomy repair (0.49%, *N*=2), new neurologic deficits (0.49%, *N*=2), and screw misplacement or screw loosening (3.4%, *N*=14). Although these authors provided an excellent, detailed assessment of their robot-related complications, it is unclear what the consequence of these errors had on other clinical outcomes, including operative time and length of stay. In addition, Keric et al. report that their study used data from two different neurosurgical departments; however, follow-up data was only reported for one hospital and the mean follow up was only 75.5 days. Furthermore, it is unclear if the rate of screw exchange included intraoperative screw revision during the index surgery.

In a single-center, retrospective analysis, Zhang et al. examined robot failure with the Renaissance system in 76 patients (874 screws) [19]. There were 39 screws (4.5%) which were adjusted during the operation, and registration failed in two patients (2.8%), both of whom had congenital scoliosis. In their multivariate analysis, they found that osteoporosis, obesity, vertebral rotation, and congenital scoliosis were independent risk factors for inaccurate pedicle screw placement (Gertzbein and Robbins—grades C, D, and E). Registration failure typically occurs when there are differences between the intraoperative positioning or imaging and the preoperative CT scan. These authors hypothesized that this can occur in patients with non-rigid scoliosis where their deformities may change after muscle relaxation with anesthesia. It is also possible that robots have more difficulty matching preoperative and intraoperative imaging in patients with poor bone mineral density. Other instances of robot failure can occur due to unreachable anatomy, which may be observed in obese patients or those with severe rotation in their vertebral bodies. Zhang et al. provided significant insight on their intraoperative robot complications and emphasized the importance of appropriate patient selection when considering robot-assisted spine surgery. However, the aim of this study was to perform risk factor analysis and the impact of these intraoperative complications on other outcomes was not addressed.

The literature on reoperations after robot-assisted spine surgery is sparse and rates are variable. Kantelhardt et al. performed a retrospective analysis on 55 patients (250 screws) who underwent robot-assisted spine surgery with a mean follow up of 3 months [7]. They reported an intraoperative complication rate of 4.7% (major hemorrhage, dural tears), wound healing issues in 13.5%, and reoperation for screw malposition in 1% of robotic-guided cases. Jiang et al examined 28 patients who underwent robot-assisted short lumbar (1 level or 2 level) fusions and reported a 30-day reoperation of 3.6% (*N*=1) [11]. Schroder and Staartjes reported on 72 patients who underwent robot-guided lumbar fusion for spondylolisthesis with a minimum 1-year follow up. None of their pedicle screws required intraoperative repositioning, and the authors denied any implant-related revisions or complications during the study’s follow-up period. The overall non-screw-related reoperation rate was 4.2% (2 patients with facet cyst removal, 1 patient with adjacent segment disease) [20]. Much of the current literature on reoperation rates after robot-assisted spine surgery is limited by short follow-up, single center series, and relatively small sample sizes.

In comparison to prior literature, this is the first multicenter study to examine robot-related complications and their potential influence on other clinical outcomes after robot-assisted spine surgery. We observed that robot complications are not uncommon (3.4%) and included screw breach, robot abandonment due to either unreachable anatomy or registration issues, and return to the operating room for screw exchange. Although robot abandonment and screw exchange, especially where there is no injury to motor/sensory function, may appear benign, these were associated with greater blood loss requiring blood transfusion and significantly longer length of hospital stay (more than 1 day), which can be costly. The reoperation rate within 1 year after the index surgery was 4.4%. The most common reasons for reoperation were wound complications (2.2%) and persistent symptoms due to inadequate decompression (1.5%). Robot factors, including robot time per screw, open vs. percutaneous, and robot system, were not statistically significant for reoperation risk. Furthermore, patients with robot-related complications at the index surgery were not at higher risk for 1-year reoperations.

Several limitations should be considered in this study. First, the minimum follow-up was 1 year after the index surgery. It is possible that complications, such as implant failure, can occur beyond this follow-up period. Second, the data collection for patient-reported outcomes was sporadic for this patient population and precluded further analysis of this important outcome variable. It is possible that patients who require multiple surgeries during the same hospital encounter or have prolonged hospital stays due to robot-related complications may have worse patient-reported outcomes. Third, cost data was not available at the time of our study and should be included in future studies to examine factors that may improve cost efficiency.

Despite these limitations, this is the first and largest multicenter study to focus on robot-assisted lumbar fusion outcomes. Our study demonstrates that robot-related complications and 1-year reoperation rates are low. Robot-related factors and complications do not increase reoperation risk; however, robot-related complications may increase the risk for greater blood loss requiring a blood transfusion and prolonged length of hospital stay. These findings can be included during the preoperative decision-making discussions with the patient.

## Data Availability

The datasets generated during and/or analyzed during the current study are available from the corresponding author on reasonable request.

## Abbreviations

CCI: Charlson Comorbidity Index
BMI: Body mass index
SD: Standard deviation
